# A Silent Obstruction: Ureteropelvic Junction Syndrome Presenting As Resistant Hypertension in an Adult

**DOI:** 10.7759/cureus.101746

**Published:** 2026-01-17

**Authors:** Miguel Martins, Valter Duarte, Daniela R Alves, Gisela Gonçalves, Ana Rita Barbosa

**Affiliations:** 1 Internal Medicine, Centro Hospitalar do Baixo Vouga, Aveiro, PRT; 2 Internal Medicine, Unidade Local de Saúde da Região de Aveiro, Aveiro, PRT

**Keywords:** nephrectomy, renal scintigraphy, secondary hypertension, thrombosed aneurysm, ureteropelvic junction, ureterovesicular junction

## Abstract

Resistant arterial hypertension warrants systematic evaluation for secondary causes, including rare structural anomalies of the urinary tract. Ureteropelvic junction obstruction can lead to chronic hydronephrosis, progressive renal dysfunction, and sustained activation of the renin-angiotensin-aldosterone system.

We present the case of a 50-year-old woman with resistant hypertension referred for secondary cause evaluation. The patient reported paroxysmal facial flushing, headaches, palpitations, and abdominal distension. Physical examination revealed a large, non-tender mass in the right upper quadrant. Laboratory workup and target organ assessment were unremarkable. Contrast-enhanced abdominal CT demonstrated severe hydronephrosis with ureteropelvic junction obstruction, marked renal parenchymal atrophy, and ipsilateral vascular anomalies with accessory renal arteries and a partially thrombosed aneurysm. Renal scintigraphy confirmed non-function of the affected kidney, and nephrectomy was proposed.

This case highlights ureteropelvic junction obstruction as a rare but clinically significant cause of secondary hypertension in adults. It emphasizes the importance of detailed imaging in evaluating resistant hypertension and the role of targeted interventions, such as nephrectomy, in selected patients, with potential improvement in blood pressure control and cardiovascular outcomes.

## Introduction

Arterial hypertension (AHT) is one of the leading causes of cardiovascular morbidity and mortality worldwide, affecting approximately one-third of the adult population. Although most cases are classified as essential hypertension, it is estimated that up to 5-10% of patients have secondary hypertension, a proportion that may be significantly higher in populations with resistant or early-onset AHT [1,2].

Resistant AHT, defined as persistently elevated blood pressure values despite the use of at least three antihypertensive agents from different classes, including a diuretic, raises particular suspicion of a secondary etiology and warrants a systematic etiological investigation [3]. Among the most frequent causes are parenchymal renal disease, renovascular hypertension, endocrinopathies, and obstructive sleep apnea [4].

Structural abnormalities of the urinary tract, namely ureteropelvic junction syndrome (UPJS), represent a rare but relevant cause of secondary AHT. Chronic obstruction may lead to hydronephrosis, progressive renal atrophy, and sustained activation of the renin-angiotensin-aldosterone system, contributing to the development or worsening of AHT [5,6]. The concomitant presence of renal vascular abnormalities, such as accessory renal arteries or aneurysms, may further aggravate renal dysfunction and increase diagnostic and therapeutic complexity [7].

## Case presentation

We present the case of a 50-year-old woman with a history of resistant AHT, referred to an Internal Medicine outpatient clinic for blood pressure control and investigation of a possible secondary cause.

The patient reported a four-month history of paroxysmal facial flushing, nausea, headaches, palpitations, and increased abdominal girth, without identifiable triggers. She denied other complaints, namely chest pain, syncope, snoring, changes in bowel habits, other skin changes, weight loss, or night sweats. Obstetric history included two previous pregnancies, one delivered by cesarean section, with the development of transient postpartum hypertension after the second delivery, which was not treated. There was no relevant family history.

She was prescribed lercanidipine 5 mg twice daily, perindopril 4 mg once daily, and indapamide 1.5 mg once daily, yet maintained ambulatory blood pressure values above 150/90 mmHg when symptomatic. On physical examination, the only remarkable finding was a large, mobile, painless mass in the right hypochondrium measuring approximately 10 × 10 cm. Body mass index was 21 kg/m², with no dysmorphic features, no palpable cervical masses, normal cardiopulmonary auscultation, no peripheral edema, and no cutaneous abnormalities. Neurological examination was unremarkable.

An initial evaluation was requested to assess target-organ damage and the most frequent causes of secondary hypertension. Laboratory tests (renal function, albumin/creatinine ratio, microalbumin/creatinine, electrolytes, thyroid function, and parathyroid hormone levels) were normal. Electrocardiogram and transthoracic echocardiography were unremarkable, and hypertensive retinopathy was excluded after ophthalmologic evaluation.

**Figure 1:**
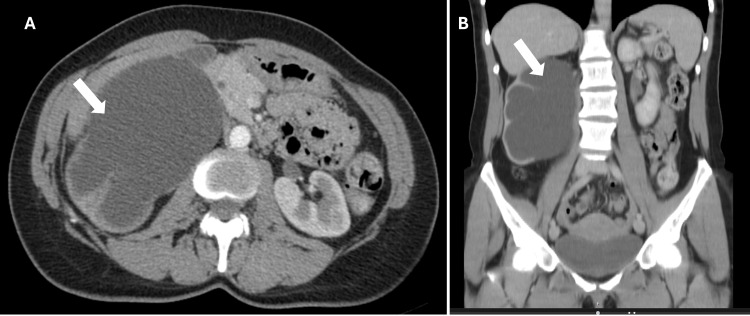
Contrast-enhanced abdominal computed tomography (A) axial and (B) coronal views Marked thinning of the right renal parenchyma and severe dilation of the pelvicalyceal system (white arrows) observed.

Contrast-enhanced abdominal computed tomography revealed marked thinning of the left renal parenchyma with severe dilation of the pelvicalyceal system, with a markedly ballooned renal pelvis and an anteroposterior diameter of approximately 85 mm, with abrupt termination compatible with probable UPJS (Figure 1).

**Figure 2:**
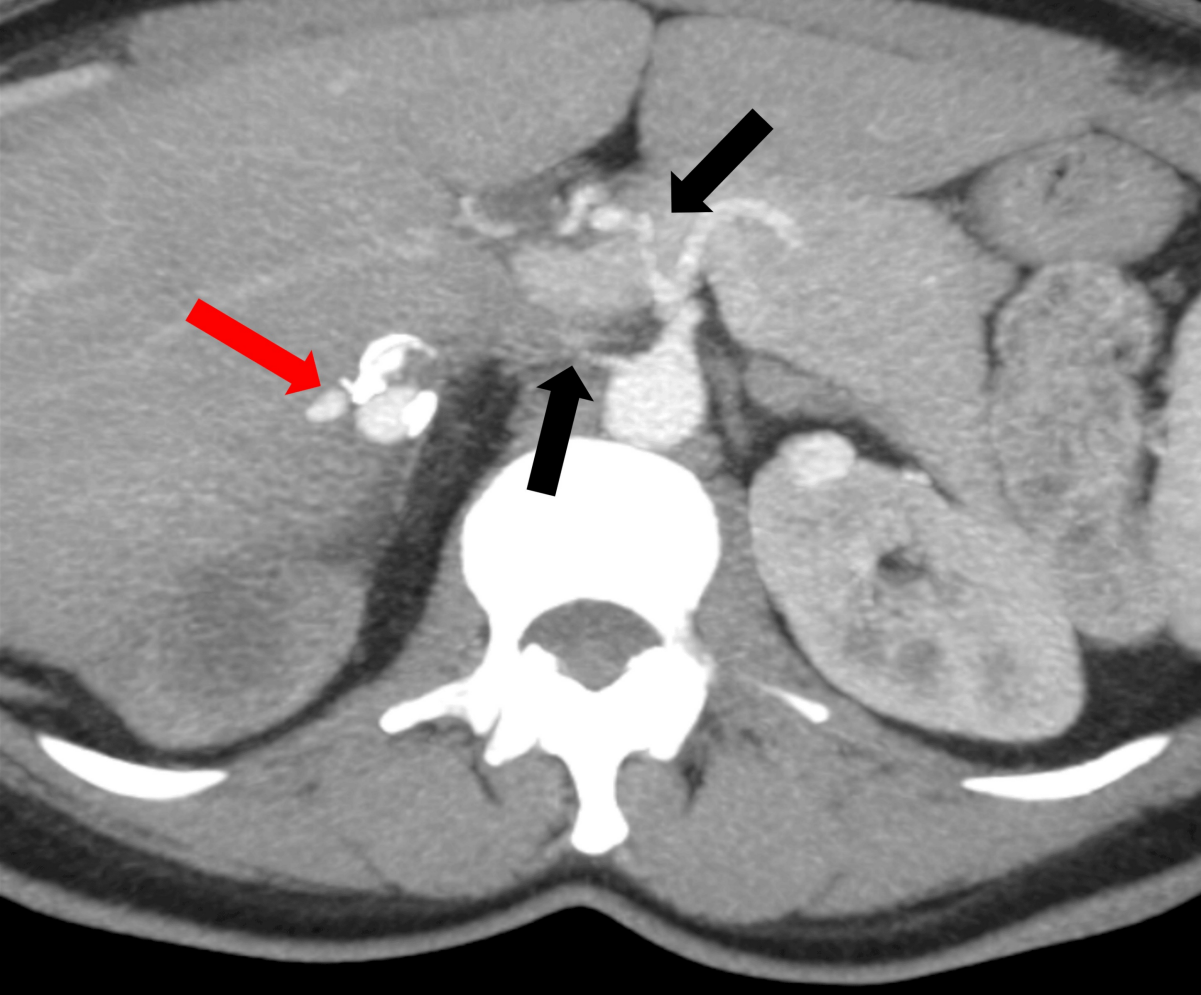
Contrast-enhanced abdominal computed tomography Two filiform renal arteries (black arrows) to right kidney, with a partially thrombosed aneurysmal (red arrow) observed.

Ipsilateral renal vascular abnormalities were also identified, including two filiform renal arteries, one arising from the celiac trunk, with a partially thrombosed aneurysmal dilation measuring 20 mm (Figure 2).

The patient was referred urgently to Urology, and renal scintigraphy demonstrated severe functional impairment of the right kidney, with a renographic pattern consistent with functional exclusion and nephrectomy was proposed.

A six-month follow-up appointment was scheduled and there was a need for titration of antihypertensive therapy to achieve adequate blood pressure control. The nephrectomy is still pending.

## Discussion

Resistant AHT is frequently associated with secondary causes, warranting systematic etiological investigation [1-3]. The present case illustrates a rare cause of secondary hypertension associated with structural renal pathology, namely UPJS with severe hydronephrosis and functional renal exclusion.

Although UPJS is more commonly diagnosed in childhood, it may present insidiously in adulthood, with hypertension representing an uncommon but clinically relevant manifestation [5]. Chronic obstruction can lead to progressive renal ischemia and sustained activation of the renin-angiotensin-aldosterone system, contributing to the development and maintenance of hypertension, even in cases of unilateral renal impairment with preserved global renal function [6].

The concomitant presence of renal vascular abnormalities, including accessory renal arteries and a partially thrombosed renal aneurysm, adds further pathophysiological complexity and may have contributed to the worsening ischemia and functional loss observed [7]. This rare association underscores the importance of detailed imaging evaluation in patients with resistant hypertension and suggestive abdominal findings.

In cases of a nonfunctioning kidney associated with difficult-to-control hypertension, nephrectomy represents a valid therapeutic option and may result in a significant improvement in blood pressure values in selected patients [5,6]. However, the response is variable and depends on the duration of hypertension and the presence of contralateral renal or vascular disease.

This case highlights the need to consider rare urological causes in the evaluation of resistant hypertension and emphasizes the role of imaging in identifying potentially treatable etiologies with a significant impact on blood pressure control and cardiovascular prognosis.

## Conclusions

Resistant AHT should prompt systematic investigation for secondary causes, including rare etiologies. This case illustrates that UPJS is an uncommon cause of secondary hypertension in adulthood, associated with severe hydronephrosis, functional renal exclusion, and concomitant vascular abnormalities, reinforcing the role of chronic renal ischemia in the pathophysiology of hypertension.

Detailed imaging evaluation is essential for the diagnosis of these conditions, enabling the identification of potentially treatable causes. In selected patients, nephrectomy may contribute to improved blood pressure control, highlighting the importance of a multidisciplinary approach in the management of resistant AHT.

## References

[1] Forouzanfar MH, Liu P, Roth GA (2017). Global burden of hypertension and systolic blood pressure of at least 110 to 115 mm Hg, 1990-2015. JAMA.

[2] Carretero OA, Oparil S (2000). Essential hypertension. Part I: definition and etiology. Circulation.

[3] Carey RM, Calhoun DA, Bakris GL (2018). Resistant hypertension: detection, evaluation, and management: a scientific statement from the American Heart Association. Hypertension.

[4] Williams B, Mancia G, Spiering W (2018). 2018 ESC/ESH Guidelines for the management of arterial hypertension. Eur Heart J.

[5] Textor SC (2017). Renal arterial disease and hypertension. Med Clin North Am.

[6] Safian RD, Textor SC (2001). Renal-artery stenosis. N Engl J Med.

[7] Krumme B, Hollenbeck M (2007). Doppler sonography in renal artery stenosis-does the resistive index predict the success of intervention?. Nephrol Dial Transplant.

